# A Review of Titanium Zirconium (TiZr) Alloys for Use in Endosseous Dental Implants

**DOI:** 10.3390/ma5081348

**Published:** 2012-08-13

**Authors:** H. Michelle Grandin, Simon Berner, Michel Dard

**Affiliations:** 1 (Independent Researcher), Calgary, T2N0K7, Canada; E-Mail: michelle.grandin@gmail.com; 2 Institut Straumann AG, Basel 4052, Switzerland; E-Mail: simon.berner@straumann.com; 3 College of Dentistry, New York University, New York, NY 10012, USA

**Keywords:** TiZr, titanium zirconium alloy, endosseous dental implant, clinical, *in vitro*, *in vivo*, reduced or small diameter implant

## Abstract

Dental implants made from binary titanium-zirconium (TiZr) alloys have shown promise as a high strength, yet biocompatible alternative to pure titanium, particularly for applications requiring small diameter implants. The aim of this review is to summarize existing literature reporting on the use of binary TiZr alloys for endosseous dental implant applications as tested *in vitro*, in animals and clinically. And furthermore to show that TiZr is “at least as good as” pure titanium in terms of biocompatibility and osseointergration. From the twelve papers that met the inclusion criteria, the current literature confirms that TiZr alloys produce small diameter implants with a strength up to 40% higher than conventional, cold-worked, grade IV titanium implants, and with a corrosion resistance and biocompatibility that is at least as good as pure titanium. The surface structure of TiZr is compatible with established surface treatments proven to aid in the osseointegration of titanium implants. Furthermore, binary TiZr alloys have been shown to achieve good osseointegration and high success rates both in animal and in clinical studies.

## 1. Introduction

Titanium (Ti) continues to be the first choice for dental implants involved in the treatment of partially or fully edentulous patients [1,2,3]. This is owing, in part, to titanium’s excellent corrosion resistance, both in air and in biological fluids, which stems from the oxide layer that forms spontaneously on its surface and, thus, renders it biocompatible. Its mechanical properties, including a high strength-to-weight ratio and ease of processing also contribute to its ubiquity in implantology. Furthermore, titanium favors osseointegration with surrounding bone tissue and it is this quality that underpins its success in both dental and orthopedic implant applications [4]. The use of commercially pure titanium (cpTi) dental implants has a long history of clinical success dating back to 1965 and Branemark’s work [5]. 

In some situations, however, the mechanical/tensile strength of cpTi is insufficient. For example, in the case when an implant is required to replace a single tooth or the implant is to be placed in a narrow edentulous ridge, small diameter implants (≤3.5 mm) are preferred [6,7,8]. Unfortunately, the reduced diameter implant has been associated with an increased risk of fatigue fracture [7,9,10]. As a result, there has been a drive to develop small diameter implants (SDIs) from titanium alloys that demonstrate improved mechanical strength. Among the Ti alloys, a titanium-aluminum-vanadium alloy known as Ti-6Al-4V, which is widely used in aerospace applications for its improved strength-to-weight ratio, has been the alloy most often used in commercial dental implants [11].

As no metal, or alloy, is entirely inert to corrosion, it is important to understand the corrosion characteristics of implant materials as well as to investigate the toxicity of any corrosion products that may result [12,13]. For instance, the biocompatibility of Ti-6Al-4V has since been called into question due to reports that the gradual release of aluminum, and particularly vanadium ions, from the surface of Ti-6Al-4V can cause local adverse tissue reactions and immunological responses [2,14,15]. Thus, the search continues for a titanium alloy with improved strength, yet lacking any toxic elements, for medical applications.

Ti-6Al-7Nb has been proposed as an alternative [16]. This alloy maintains the strength-inducing microstructure of Ti-6Al-4V with its Al-stabilized α-phase and replaces the V-stabilized β-phase with a non-toxic Nb-stabilized β-phase. Although this αβ structure lends to the strength of the biomedical alloy, it is also known to present different etching mechanisms than cpTi, a property which will be discussed below in reference to the importance of modifying surface roughness for improved osseointegration [17]. Further Ti alloys, incorporating non-toxic elements such as zirconium (Zr), niobium (Nb), tantalum (Ta), palladium (Pd) and indium (In), are also being explored for their ability to match the mechanical strength and corrosion resistance of Ti-6Al-4V, with improved biocompatibility [13,18,19]. In particular, alloys containing zirconium have demonstrated both the required mechanical strength and a high resistance to corrosion in biological fluids [13,17,19]. The biocompatibility of TiZr-based alloys indicates an improvement even over cpTi, the gold standard [20,21]. 

As alluded to above, in addition to the selection of implant material, the surface topography and chemistry have proven to be important aspects in determining implant success. The positive influence of surface topography on osseointegration, achieved through reducing techniques such as grit-blasting, sandblasting and acid-etching, has been the topic of investigation for several decades. Today, microrough surfaces dominate the market as they have been shown to achieve faster bone integration, a higher percentage of bone to implant contact (BIC) and a higher resistance to shear, as determined by removal torque values (RTVs), when compared with titanium implants with a polished or machined surface [22]. A combination of sandblasting and acid-etching of titanium implant surfaces, referred to as SLA^®^ surface treatment (Institut Straumann AG, Basel, Switzerland), has been tested both in animals and clinically, with results confirming the ability for the topography to enhance bone integration and long term stability [23,24,25,26,27]. 

Furthermore, it has been demonstrated that increased surface energy can influence the biological activity of cells *in vitro*, and in particular, hydrophilic surfaces can be osteogenic, thus influencing bone cell maturation and differentiation [28,29]. Correspondingly, a hydrophilization technique has been added to the SLA treatment mentioned above in order to increase surface energy and it is termed an SLActive^®^ surface treatment (also known as modSLA in the literature) (Institut Straumann AG, Basel, Switzerland) [30]. This treatment, which involves rinsing the micro-roughened surfaces under nitrogen and storing them in a saline solution instead of air, has been shown to improve initial wetting conditions by lowering contaminations and by retaining a more activated Ti surface. The beneficial effects on cell differentiation and growth factor production of SLActive *versus* SLA surface-treated Ti implants have been documented *in vitro*, in animal and in clinical studies [31,32,33]. Interestingly, in addition to increased wettability, there is some indication that the nanostructures that spontaneously form on SLActive surfaces may also contribute to its stronger bone response [34]. 

In exploring new alloys with improved mechanical strength, it would be optimal if one could also conserve the micro-roughened topography and hydrophilic surface properties that have proven to be integral to the success of cpTi implants. In this respect, the binary titanium zirconium (TiZr) alloy stands apart from any of the αβ structured alloys in that it maintains the same α structure as cpTi and is compatible with both the SLA and the SLActive treatments [17]. As a result, TiZr presents itself as an attractive implant material, particularly for use in small diameter implant (SDI) applications, owing to its improved strength, while maintaining the biocompatibility and osseointegration properties observed for cpTi. 

Therefore, the aim of this review is to summarize the existing literature, dating back to 1987 and forward to April 2012, reporting on the use of binary TiZr alloys for endosseous dental implant applications as tested *in vitro*, in animal and in clinical studies. And furthermore, to show that TiZr is “at least as good as” the gold standard, cpTi, in terms of biocompatibility and osseointegration. 

## 2. Methods

In order to analyze all relevant literature for *in vitro*, animal and clinical studies of binary TiZr alloys, a literature search was performed, using a systematic approach, for the time period of 1987 to April 2012. Within the PubMed search engine, the following three key word strings were utilized: (1) titanium zirconium (dental OR implant) NOT zirconia; (2) TiZr; and (3) Titanium-Zirconium. The inclusion criteria for this search were: (1) studies that examine binary titanium zirconium alloys as an alternative dental implant material; (2) studies that test the TiZr alloy in clinical, in animal and/or *in vitro* settings; (3) studies written in English, during the time period of 1987 to April 2012. The exclusion criteria were: (1) studies examining ternary or higher order TiZr Alloys, *i.e.*, alloys including additional elements such as Nb, Ta, *etc.*; (2) studies examining TiZr alloys for applications in non-endosseous dental applications such as wires, crowns, *etc.*; (3) research on ceramic zirconia; and (4) studies examining additional coatings on TiZr implants. Electronically available abstracts were first scanned to remove any articles that clearly did not meet inclusion/exclusion criteria and the full texts were obtained for the remainder of the articles. The full texts were then used to reassess inclusion/exclusion criteria. Additional relevant articles were included when found referenced within the articles retrieved through the PubMed search.

## 3. Results 

The PubMed search using the key word string “titanium zirconium (dental OR implant) NOT zirconia” resulted in 113 articles. Of these, the total number that met the inclusion criteria for this review was eight. An additional paper was found using the search “TiZr”. The third search added no additional papers. However, an additional 3 were added as they were discovered during the analysis of the search retrieved papers. From the 12 articles that met the criteria, four were dedicated to *in vitro* and/or mechanical studies of TiZr [17,19,29,35], five articles investigated the performance of TiZr implants in animals [21,36,37,38,39], while three articles analyzed clinical outcomes of TiZr dental implants [6,40,41]. The results of these studies are summarized in Table 1, Table 2 and Table 3.

**Table 1:** *In vitro* studies of TiZr dental implants.

First Author, Year	Type of TiZr	Study Details	Main Results
Sista *et al.*, 2011 [29]	TiZr (50% Zr)	TiZr compared with cpTi and TiNb, cell assays using mouse osteoblast cell line (MC3T3-E1) and surface characterization.	More cells attached to TiZr, than Ti or TiNb, although spreading was similar. TiZr and Ti showed higher ALP activity and OC expression than TiNb, indicating differentiation of cells.
Bernhard *et al.*, 2009 [17]	TiZr * (13%–17% Zr), SLActive^®^	TiZr compared with cpTi, cell assays using MG-63 osteoblast-like cells.	TiZr is compatible with SLActive surface treatment used for cpTi. TiZr was found to be at least as biocompatible as cpTi. Tensile and 0.2% yield strength were about 40% and 60% higher than that of cpTi, respectively.
Zhang *et al.*, 2009 [19]	TiZr (12% Zr)	TiZr compared with cpTi, Ti6Al4V, TiAlMoZr, TiNbTaZr and stainless steel, cell assays using human lymphoid cells (CEM) and MC3T3-E1 cells.	Zr-containing Ti-alloys demonstrated highest corrosion resistance in the presence of cells and various electrolytes.
Kobayashi *et al.*, 1995 [35]	TiZr (0%–100% Zr)	TiZr compared with Ti-6A1-4V and Ti-Zr-6Al-4V.	Hardness and tensile strength increased with % Zr to a maximum of about 2.5 times cpTi at 50% Zr. It was also found to increase hardness in TiZrAlV over the standard TiAlV.

* Commercially known as Roxolid^®^ (Institut Straumann AG, Basel, Switzerland) small diameter implants.

**Table 2:** Animal studies of TiZr dental implants.

First Author, Year	Type of TiZr	Study Details	Main Results
Saulacic *et al.*, 2012 [37]	TiZr * (15% Zr), SLActive^®^	TiZr compared with cpTi and Ti6Al4V, in minipigs, at 1, 2, 4 and 8 weeks.	Comparable bone-to-implant-contact (BIC) values were observed for TiZr and Ti, increasing from 2 to 8 weeks. In contrast, BIC values were significantly lower for TiAlV.
Gottlow *et al.*, 2011 [36]	TiZr * (13%–17% Zr), SLActive^®^	TiZr compared with cpTi, in minipigs, at 4 weeks.	Removal Torque (RT) values were higher and bone area larger for TiZr. BIC was similar for cpTi and TiZr.
Thoma *et al.*, 2011 [39]	TiZr* (15% Zr), SLActive^®^	TiZr compared with cpTi, in dogs, at 2, 4, and 8 weeks.	Uneventful and safe healing was observed for both TiZr and Ti implants. No statistical difference between BIC at any time point, which were all about 80%.
Ikarashi *et al.*, 2005 [21]	TiZr (50% Zr)	TiZr compared with Ti and Cr plates, in rats, at 8 months.	TiZr alloy shows better biocompatibility and mechanical properties than cpTi.
Shibata *et al.*, 1987 [38]	Porous TiZr (40% Zr), using 3 different particle sizes	In rabbits, at 2, 4, 8, and 12 weeks.	Sintering of larger particle sizes led to higher bone ingrowth, bone filling and higher bonding strength (as determined by push-out tests).

* Commercially known as Roxolid^®^ (Institut Straumann AG, Basel, Switzerland) small diameter implants.

**Table 3:** Clinical studies of TiZr dental implants.

First Author, Year	Type of TiZr	Study Details	Main Results
Al-Nawas *et al.*, 2011 [6]	TiZr * 13% Zr), SLActive^®^, 3.3 mm diameter	TiZr compared with Ti Grade IV 3.3 mm diameter, 87 patients with removable overdentures, at 6 and 12 months.	At 12 months, TiZr performs at least as well as Ti Grade IV, as determined by bone level change, plaque and sulcus bleeding. Implant success rates were 96.6% and 94.4% for TiZr and cpTi, respectively.
Barter *et al.*, 2011 [40]	TiZr * (13%–15% Zr), SLActive^®^, 3.3 mm diameter, splinted to regular Ti implant	TiZr compared with Ti Grade IV 3.3 mm diameter, 22 patients, at 2 years.	This paper reports the first clinical test of reduced diameter TiZr and validates proof of concept, as all 20 implants were considered successful after 2 years. Bone level change (<1 mm), and probing pocket depth meet established success and survival criteria.
Chiapasco *et al.*, 2011 [41]	TiZr * (13%–17% Zr), SLActive^®^, 3.3 mm diameter	Studied prosthetic loading, in 18 patients, at 0 and 2–12 months.	All implants successfully osseointegrated and completed prosthetic rehabilitation. Peri-implant bone resorption ranged from 0 to 1 mm. Survival and success rates were 100%.

* Commercially known as Roxolid^®^ (Institut Straumann AG, Basel, Switzerland) small diameter implants.

## 4. Discussion

### 4.1. Mechanical Properties and in Vitro Results

The need for increased mechanical strength from biomaterial implants, both in dental and orthopedic applications, has motivated the search for Ti-alloy alternatives that are free of toxic elements such as vanadium. In dentistry, the use of small diameter implants would be advantageous in situations where single teeth are being replaced or when implants need to be placed within narrow edentulous ridges. However, their use has so far been limited to the “esthetic zone”, due to risk of fatigue fracture under high loading. A higher mechanical strength Ti-alloy for small diameter implants would obviate the need for reconstructive surgery and/or bone grafting or augmentation in the narrow ridges. The titanium-zirconium (TiZr) alloys present as a promising candidate for such applications.

In 1995, Kobayashi *et al.* proposed titanium zirconium alloy as a base for future biomaterials, owing to its increased hardness and tensile strength, while maintaining the corrosion resistance and biocompatibility of pure titanium [35]. In their work, the tensile strength of alloys containing 25%–75% zirconium was found to be 2.5–3 times higher than either pure titanium or pure zirconium. The superior corrosion resistance of zirconium-containing titanium alloys was demonstrated both in serum and in the presence of cells by Zhang *et al.* [19]. In 1997, Steinemann proposed a binary TiZr alloy for surgical implants [42]. The work of Steinemann was further developed, and in 2009 Bernhard *et al.* published the findings on the binary TiZr alloy, called Roxolid^®^ (Institut Straumann AG, Basel, Switzerland), which contained 13%–17% Zr, for use in dental implants [17]. In this work, the tensile strength of TiZr, with a value of 953 MPa, was found to be approximately 40% greater than that of the minimum requirement for cpTi implants, *i.e.*, that of cold worked, grade IV cpTi, which has a value of 680 MPa (see also Table 4) [43]. Furthermore, this value of 953 MPa reported for TiZr tensile strength is on par with, and even somewhat higher than, the values of 860 MPa [44] reported for Ti-6Al-V and 900 MPa [45], reported for Ti-6Al-7Nb. Surface characterization confirmed the compatibility of TiZr with the SLActive surface treatment that currently aids in the osseointegration of certain commercial cpTi implants by introducing micro (and nano) roughness as well as introducing a strong hydrophilicity. This is a result of the fact that TiZr takes on the same α-phase crystal structure of Ti. In the work of Bernhard *et al.*, the fatigue behavior was also tested and it was found that the endurance levels of TiZr implants are 13%–42% higher compared to cold-worked cpTi implants with the same length and diameter, depending on the different implant/abutment configurations.

**Table 4:** Comparison of TiZr *vs.* Ti tensile strength, removal torque values (RTV), bone-to-implant contact (BIC), bone level change, and implant success rates.

Material	Study	Subject	Follow up time	Tensile Strength	RTV	BIC	Bone level change	Success Rate
Ti *	Al-Nawas 2011 [6]	Humans	–	–	–	–	−0.3 mm +/− 0.6 mm	94.4%
	N/A	–	–	–	–	–	–	–
	N/A	–	–	–	–	–	–	–
	Saulacic 2012 [37]	Minipigs	8 weeks	–	–	84.67%	–	–
	Gottlow 2011 [36]	Minipigs	4 weeks	–	204.7 ± 24.0 N cm	72.3 ± 20.5%	–	–
	Thoma 2011 [39]	Dogs	8 weeks	–	–	83.4% ± 5.9%	+0.02 mm ± 0.33 mm	–
	Bernhard 2009 [17]	*In vitro*	–	680 MPa	–		–	–
TiZr *	Al-Nawas 2011 [6]	Humans	1 year	–	–	–	−0.3 mm ± 0.6 mm	96.6%
	Barter 2011 [40]	Humans	2 years	–	–	–	−0.33 mm ± 0.54 mm	95.2%
	Ciapasco 2011 [41]	Humans	3–19 months	–	–	–	in range of 0–1 mm	100%
	Saulacic 2012 [37]	Minipigs	8 weeks	–	–	74.50%	–	–
	Gottlow 2011 [36]	Minipigs	4 weeks	–	230.9 ± 22.4 N cm	70.2% ± 17.3%	–	–
	Thoma 2011 [39]	Dogs	8 weeks	–	–	86.9% ± 6.8%	−0.09 mm ± 0.33 mm	–
	Bernhard 2009 [17]	*In vitro*	–	953 MPa	–	–	–	–

* Both Ti, Grade IV and TiZr (13%–17% Zr), Roxolid^®^ had SLActive^®^ surface treatment in all studies listed here.

*In vitro*, the work of Sista *et al.* has demonstrated that MC3T3-E1 osteoblasts attach in higher number to TiZr (50% Zr), compared with Ti and TiNb (50% Nb), and that the attached cells exhibit higher alkaline phosphatase (ALP) activity and osteocalcin (OC) expression on TiZr after 7, 15 and 21 days [29]. Also of interest is the work of Chen *et al.* that detailed the bioactive nature of TiZr, wherein alkali and heat treated TiZr (50% Zr) as well as cpTi, with a mean roughness (Ra) of 0.6 µm, elicited the formation of a dense, uniform and continuous layer of apatite when submerged in simulated body fluid (SBF) [46]. Positive results have also been reported for tertiary or higher-order zirconium-containing Ti-alloys, such as those studied by Kim *et al.*, contained Nb, Ta, Pd and In, in addition to TiZr (roughly 20% Zr) [18]. These experimental TiZrNbTaPd(In) alloys were found to have roughly twice the hardness (300 MPa *vs.* 166 MPa) and tensile strength (967 MPa *vs.* 512 MPa) of Ti, while eliciting similar cell growth and cell attachment patterns as Ti after 2 and 7 day incubation periods. Together, the *in vitro* work confirms both the biocompatibility and the bioactive nature of TiZr surfaces. In the future, it would be of interest to see the results of longer term investigations of TiZr corrosion in order to determine if there is potential for allergic and immunogenic reactions after prolonged exposure.

### 4.2. Results of Animal Studies

Early work by Ikarashi *et al.* investigating the performance of TiZr in animals further illustrated its biocompatibility by comparing TiZr, cpTi and chromium (Cr) plates implanted in rats, with the finding that TiZr performed better, even, than pure Ti or Cr [21]. Prior to that, in 1987, a study by Shibata *et al.* found that the sintering of larger particle sizes in the formation porous TiZr (40% Zr) implants led to a higher bone ingrowth, when implanted in rabbits, and higher bonding strength as determined by push-out tests [38]. 

More recently, there have been a few animal studies comparing the osseointegration and stability of commercially available 3.3 mm diameter TiZr implants (Roxolid^®^, Institut Straumann AG), containing 13%–17% Zr, with cpTi (see Table 4 for cross comparison). Both the TiZr implants and the grade IV cpTi are subject to the same SLActive surface treatment, which introduces both a micro-roughness and a hydrophilicity to the surface. The results of Thoma *et al.* found that both TiZr and Ti implants had comparable and uneventful safe healing when implanted in dogs after 2, 4 and 8 weeks. The bone-to-implant contact (BIC) was not found to be statistically different one from the other, with a value of approximately 80% for both TiZr and Ti (see also Table 4) [39]. A study by Gottlow *et al.* further confirmed that TiZr is at least as good as Ti in terms of osseointegration after 4 weeks in minipigs, with BICs of about 70% for both materials (see Table 4). However, bone area was actually larger around the TiZr implants [36]. Removal torque values were statistically higher for TiZr (Table 4) indicating a higher stability around TiZr implants. Saulacic *et al.* also compared TiZr to cpTi and Ti-6Al-4V in minipigs after 1, 2, 4 and 8 weeks [37]. The BIC was again found to be statistically equivalent between TiZr and cpTi, while superior to Ti-6Al-4V. In addition, multinucleated giant cells were found on Ti-6Al-4V implants, but not on TiZr or cpTi after 8 weeks. 

Together, the results of the animal studies performed to date confirm that small diameter (3.3 mm) TiZr dental implants perform at least as well as cpTi in animals in these short term studies. The work of Saulacic *et al.* also highlights the superiority of TiZr *versus* Ti-6Al-4V in terms of bone-to-implant contact and biocompatibility as seen by the number of multinucleated giant cells found on Ti6Al4V implant surfaces. Furthermore, the work by Gottlow *et al.* indicates that TiZr has a stronger osseointegration than cpTi as seen by the higher removal torque values and the higher bone area found around TiZr implants after 4 weeks. Thus, it appears that TiZr may even be a better material than pure titanium for use in dental implants; however, long-term studies are required.

### 4.3. Clinical Results

Three clinical studies investigating the commercially available TiZr implants (Roxolid^®^, Institut Straumann AG) with respect to grade IV cpTi have also been carried out (see Table 4 for cross comparison). Again, both implant types are subjected to the same SLActive surface treatment. Chiapasco *et al.* followed 51 TiZr implants in 18 patients to evaluate the success rates of the small diameter implants (SDIs) in horizontally deficient ridges [41]. All implants achieved osseointegration and completed the prosthetic rehabilitation; therefore a 100% success rate was realized (Table 4). Peri-implant bone loss ranged from 0 to 1 mm at the end of the observation period (range: 3–19 months). In a study by Barter *et al.*, 22 patients received one 3.3 mm TiZr implant with a regular neck standard plus design which was splinted to a standard grade IV cpTi regular neck implant with a fixed prosthesis [40]. Twenty of the 22 patients had a successful and surviving implant at the 2 year follow-up. The mean change in functional bone level at 2 years was −0.33 mm ± 0.54 mm (Table 4). It was concluded that the new implants meet established success and survival criteria after 2 years. In a double-blind randomized controlled trial of TiZr *vs.* cpTi small-diameter bone level implants by Al-Nawas *et al.*, it was found that TiZr SDI provide at least the same outcomes as grade IV cpTI after 12 months [6]. Each of 91 patients received one TiZr and one Ti implant in the interforaminal region. The peri-implant bone level change was −0.3 mm ± 0.5 mm and −0.3 mm ± 0.6 mm for TiZr and Ti, respectively, while success rates were 96.6% and 94.6%, respectively (Table 4). Interestingly, in another study, it has also been shown that TiZr, much like Ti, is compatible with a procedure for guided bone growth around implants with the use of BMP-2 and scaffolds [47].

The results of these clinical studies [6,40,41] certainly highlight the high success rate of TiZr implants in patients (ranging from 95.2% to 100%). The TiZr implants meet established success and survival criteria for dental implants by demonstrating good osseointegration, with acceptable bone-level changes, probing pocket depth, plaque and sulcus bleeding. Based on these preliminary results, the performance of the TiZr implant material is deemed to be safe and reliable. However, longer terms studies investigating TiZr as an implant material as well as the success of small diameter implants in general are necessary prior to finding non-restrictive clinical recommendation for their use.

## 5. Conclusions 

The current literature reveals that titanium zirconium (TiZr) alloys are at least as biocompatible as pure titanium and in some cases appear to have superior biocompatibility to cpTi. Furthermore, the corrosion resistance of TiZr is as good as, if not better than, cpTi, while its α-phase crystal structure ensure that the surface treatments that have been successful for enhancing the osseointegration of Ti implants can continue to be implemented with the newer TiZr alloy. Finally, the strength of TiZr implants is found to be up to 40% higher than the strength of cold-worked, grade IV Ti, which makes TiZr a great candidate for small diameter implants, even in high loading situations. To date, there has only been a handful of animal and clinical studies testing the performance of reduced diameter TiZr implants (3.3 mm) *in vivo*. From these studies, the results unanimously indicate that the TiZr implants perform at least as well as grade IV Ti implants. Good osseointegration and high implant success rates have been observed for TiZr in each study. There remains a need to follow the TiZr implants over a longer time period, as 2 years is the longest follow-up reported to date. In conclusion, TiZr has demonstrated its ability to perform at least as well as cpTi *in vitro*, in animals and clinically, thus underlining its great potential to become a dominant implant material of the future.
